# Copper Oxide Nanoparticle-Decorated Carbon Nanoparticle Composite Colloidal Preparation through Laser Ablation for Antimicrobial and Antiproliferative Actions against Breast Cancer Cell Line, MCF-7

**DOI:** 10.1155/2022/9863616

**Published:** 2022-03-08

**Authors:** Salman A. A. Mohammed, Khawla S. Khashan, Majid S. Jabir, Farah A. Abdulameer, Ghassan M. Sulaiman, Mohsen S. Al-Omar, Hamdoon A. Mohammed, Aseel A. Hadi, Riaz A. Khan

**Affiliations:** ^1^Department of Pharmacology and Toxicology, College of Pharmacy, Qassim University, Qassim 51452, Saudi Arabia; ^2^Division of laser Sciences and Technology, Department of Applied Sciences, University of Technology, Iraq; ^3^Division of Biotechnology, Department of Applied Sciences, University of Technology, Baghdad, Iraq; ^4^College of Health and Medical Techniques, Gilgamesh Ahliya University, Baghdad, Iraq; ^5^Department of Medicinal Chemistry and Pharmacognosy, College of Pharmacy, Qassim University, Qassim 51452, Saudi Arabia; ^6^Medicinal Chemistry and Pharmacognosy Department, Faculty of Pharmacy, JUST, Irbid 22110, Jordan; ^7^Department of Pharmacognosy, Faculty of Pharmacy, Al-Azhar University, Cairo 11371, Egypt

## Abstract

Copper oxide (CuO) nanoparticle- (NP-) decorated carbon NPs (CNPs) were produced as colloidal suspension through pulsed laser ablation technique in liquid (PLAL) medium. The antimicrobial activity of the produced NPs was tested against *Staphylococcus aureus* (*S. aureus*) and *Escherichia coli* (*E. coli*), and anticancer activity was tested against breast cancer cell line, MCF-7, together with the biocompatibility assessment of these NPs. The X-ray diffraction (XRD) patterns of the obtained CNPs showed peaks at 26.58° and 43.78° (2*θ*) identical to (002) and (111) planes, respectively, of the carbon phases. It also displayed new peaks at 38.5° and 48.64° (2*θ*) after doping with CuO NPs. Transmission electron microscope (TEM) images revealed the crystalline nature with the spherical shape of the prepared CNPs with 5-40 nm diameter ranges. In addition, the NP effects on the bacterial cell walls and nucleic acid were confirmed using a scanning electron microscope (SEM) and microscopic fluorescence analysis. The NPs showed antibacterial activity through SEM examinations against the pathogenic microbial species, *S. aureus* and *E. coli*. In the cellular material release assay, the optical density of the bacterial cells, treated with NPs, displayed a significant increase with the time of exposure to NPs, and the cytotoxicity reached more than 80% of the level for the CNPs decorated with CuO NPs. The morphology of the MCF-7 cells treated with NPs decreased numbers, and the loss of contact with the surrounding cells was observed. These results confirmed that the CNPs decorated with CuO NPs have no observable side effects and can be safely used for therapeutic applications. It is also noteworthy that it is the first report of preparation of CuO NPs decorated with CNPs (CuO NPs-CNPs) by PLAL, and the produced NPs showed antimicrobial antiproliferative activities against breast cancer cell lines, MCF-7. The main advantage of the PLAL technique of synthesizing CuO NPs-CNPs provided a two-step, cost-effective, and eco-friendly method.

## 1. Introduction

Cancer is an uncontrolled growth of cells causing health concerns globally. Chemotherapy is one of the available methods for cancer treatment, which is expensive with harmful side effects. Therefore, there is an urgent need for effective, inexpensive, and nontoxic treatments with minimal side effects [1–3]. Nanomaterial structures were reported to have useful applications in cancer diagnosis and therapy [4, 5]. Carbon nanoparticles (CNPs) have attracted attention in different fields due to their unique properties, like a nontoxic, biocompatible, and photochemical stability [6]. Therefore, the surface modification of CNPs via decorating with metal (Ag, Au, and Pt) and metal oxide nanoparticles (ZnO, Fe_2_O_3_, and TiO_2_) is the most functional methods to enhance the antibacterial performance of and reduce the adverse effects of the metal and metal oxide materials. Recently, the development of carbon nanostructures decorated with different types of NPs demonstrates a promising process for different applications [7–10]. Among them, copper materials demonstrated the ability to modify cancer cell metabolism and induce cell death. Furthermore, studies on utilizing these nanostructures reported the production of reactive oxygen species (ROS) and DNA damage. Therefore, transferring copper ions to the selected tissues with high efficiency and the lowest loss levels is significant. There is a potential to prepare a new nanosystem, where the components are loaded on a carrier to convey ions, and CNPs are demonstrated to be a good carrier [11]. Different ways are used to produce nanostructured materials [12–15]; among them, pulsed laser ablation in liquid (PLAL) is the best method for synthesizing different types, sized, and shaped nanomaterials, in addition to other advantages like cost control and without the production for any by-product(s) and purification [16–24]. In addition, the NP morphology is prone to modification by using different liquids and laser parameters. In addition, there is no necessity for a vacuum system during preparation [11], and the method is utilized to produce NPs of the desired composition with chemical-free pollution [24]. One of the advantages of the PLAL method is that the produced NPs have a narrow size distribution as homogeneous nucleation can be saturated and quickly reduced to smaller sizes in the colloid [25]. Despite improvements in nanoscale technology, there is insufficient information on the negative impacts of nanomaterials on health, and extensive nanotoxicology assessments are well anticipated. Nanoscale particle properties have important effects on toxicity [26]. The NP-conjoined nanomedicine provides platform for treating cancers and bacterial infections through their potential in inhibiting biofilm formation, cell entry, intracellular retention, and augmentation of antimicrobial agent potency [27, 28]. NPs gather at infection zone due to their size, surface charge, and the outsized volume to area ratio.

The mechanism of generation of NPs in PLAL can be briefed as follows: the laser beam hits the surface of the target; a plasma plume is created due to the high energy transferred to the material. This plume in liquid environments confines the plasma, increasing the temperature, pressure, and density to a thermodynamic state where particular reactions can occur between the plasma produced by the target material and the liquid molecules, allowing for the production of metastable structures and particles with varying compositions. During PLAL process, the obtained material interacts with liquid molecules in 3 phases, i.e., intersection of ablation plume with surrounding fluid, interaction with cavitation bubble of the gas phase, and the adjoining fluid at ambient temperature and pressure postcavitation bubble collapse. Oxygen atoms originating from the water molecules interact with the ablated target species, depending on the present ROS concentration and the metal molecules' redox potential [29, 30]. The primed NPs can potentially augment their payload through their dynamic movement to host and bacterial cell receptors. Several antimicrobial agents tagged with nanocarriers demonstrate their potential against sensitive and resistant bacterial strains and decrease the drug's side effects [31–36]. Herein, the CuO NP-decorated CNPs were prepared using the PLAL, and *in vivo* side effects as antimicrobial agent against *Staphylococcus aureus* (*S. aureus*) and *Escherichia coli* (*E. coli*) and anticancer activity against human breast adenocarcinoma (MCF-7) cell lines were investigated. The current work is primarily driven by the environmental concerns to develop a green route for NP production, which is also contamination-free and provides opportunities to develop therapies against cancer in general and breast cancer in particular. The method also can open up possibilities of NPs preparation from different target materials for several biomedical applications for *in vivo* evaluations.

## 2. Materials and Methods

### 2.1. Cells and Reagents

MCF-7 breast cancer cells were procured from Iraqi Center for Cancer and Medical Genetics Research. Acridine orange, ethidium bromide, trypsin–EDTA, fetal bovine serum, 3-(4,5-dimethyl thiazyl-z-yl)-2,5-diphenyl tetrazolium (MTT), and crystal violet stain were procured from Sigma-Aldrich (St. Louis, MO, USA), while RPMI-1640 medium was obtained from Gibco (USA). All other chemicals and reagents were of analytical grade level.

### 2.2. Preparation of Colloidal NPs

Two steps were followed to produce CNPs decorated with CuO NPs; the first step was the synthesis of CNPs using Nd : YAG laser (1064 nm; 1 Hz, 9 ns) ablation of graphite pellets (99.9%), set in a bottom of a vessel containing 3 ml of deionized water (DIW), with a fluence of 10.6 J/cm^2^. In the second step, suspension of copper (99.9%) with CNPs produced at the same fluency and ablation formed CNPs decorated with CuO NPs.

### 2.3. Characterization of Colloidal NPs

The X-ray diffraction characterization was performed utilizing a Cu-K*α* source (*λ* = 1.54060 Å; Shimadzu model). The morphological features and chemical composition were described using a transmission electron microscope (TEM, Philips model) and energy dispersive spectroscopy (EDS), respectively.

### 2.4. Antibacterial Activity of NPs

The gram-negative and gram-positive bacterial strains *Escherichia coli* (*E. coli*) and *Staphylococcus aureus* (*S. aureus*), respectively, were investigated for the antibacterial potential of the NPs. The bacterial strains were cultured at 37°C on M–H agar plates, and inoculations were collected from the freshly cultured plates into tubes comprising nutrient broth (NB, 50 ml) for evaluation of NP effect on the bacterial growth curve. The bacterial species were expanded till NB optical density (OD) at 600 nm reached 0.1, equivalent to 108 (CFU/ml) bacterial concentration. The bacteria were then applied to fresh NB (50 ml) supplemented with NPs (50 *μ*g/ml) and incubated for 24 hours at 37°C with mild shaking. A spectrophotometer measured the OD of the NB for 12 hours, assessing bacterial growth.

### 2.5. Release of Cellular Materials

The investigation of the release of cellular materials by the treated organisms was performed using sterile peptone water. The sterile peptone water was seeded with gram-positive and gram-negative bacterial strains at 1 × 10^6^/ml density and incubated for 24 h. After that, the prepared bare carbon and carbon–CuO at 50 *μ*g/ml concentration were added to the media. After a determined time (30, 60, 120, 180, and 240 min) posttreatment, the peptone media were centrifuged at 3500 rpm to obtain clear supernatant. The absorbance of the obtained clear supernatant was determined at 260 nm. The investigation results were presented as absorbing material percentage for each interval at 260 nm with the indicated time [24].

### 2.6. Study of Bacterial Strain Shape using SEM

Morphological alternations in *E. coli* and *S. aureus* were observed using a scanning electron microscope (SEM) according to the method described by Khashan et al. [37]. Briefly, the prepared bacterial suspensions were loaded on silicon wafer slide and fixed on the SEM stubs. The slides were coated with gold film (5 minutes), a gold thin film (approximately 20 nm) was spread on the cell surface, and cells were examined using SEM (TESCAN, Vega III, Czech Republic) [38].

### 2.7. Detection of Live/Dead Bacterial Strain

Antibacterial activity of the NPs was quantified using a fluorescent microscope. Orange-ethidium bromide (AO/EtBr) method was employed for cell viability. A thin film of mixture (50 *μ*l bacterial suspension (treated/untreated) and 50 *μ*l (10 *μ*g/ml AO/EtBr stock solution)) was coated onto a glass slide and visualized using immunofluorescent microscope. The AO (green) and EtBr (red) stained the living and dead cells, respectively.

### 2.8. MTT Assay

The MTT assay was performed according to the previously described procedure by Khashan et al. [37]. Cells were seeded at densities of 1 × 104 cells/ml in 96-well microliter plates covered with RPMI medium and left overnight, then NPs were added in triplicates and left for 72 h followed by cell marking with MTT and later washed 3x using PBS. The crystal residuals were dissolved in DMSO, and a microplate reader evaluated the absorbance at 492 nm. To display the cells' shape, the wells were recolored with crystal violet and left for 15 min, followed by washing and images obtained using an inverted microscope at 40x.

### 2.9. Live-Died Double Stain Assay

This assay was used to test the ability of NPs to induce apoptosis in MCF-7 cells. The cell was seeded at a 1 × 105 cells/ml concentration, and then, NPs were added and left for 48 h. Subsequently, they were rinsed 3 times with PBS, and then, the AO/EtBr dye was added. After 2 min, the samples were examined by fluorescent microscopy with the scale bar 10 *μ*m.

### 2.10. Flow Cytometry

Apoptosis detection using flow cytometry was done according to the previous protocol [39]. The MCF-7 cells were treated with NPs and then analyzed by determining the ratio of cells with nucleus concentration and fragment. The cells were seeded for 24 h and then treated with prepared NPs. Cells were suspended in the FACS buffer followed by staining with annexin V-FITC (Invitrogen, Carlsbad, CA) and measured to investigate the apoptotic cells using flow cytometer assay.

### 2.11. Side Effects of Prepared NPs

To investigate the *in vivo* toxicity of the CNPs decorated with CuO NPs, 15 male mice weighing between 30-42 g were maintained under constant environmental conditions. The institutional Research Ethics Committee, College of Pharmacy, Qassim University, Saudi Arabia, approved the animal experimental procedure and care (Approval ID 2018 - CP-2). Animal groups (5 mice/group) involved were the following: control group did not receive any treatment, while the 2^nd^ and 3^rd^ groups were injected with CNPs and CNPs decorated by CuO NPs, respectively. The experiments were conducted for four weeks. The blood samples from anesthetized animals were collected via heart puncture and then centrifuged for 10 min at 5000 rpm. The kidney functions like urea and creatinine were estimated using the enzymatic method. For the histological examination, the tissue was washed with PBS and fixed in 10% formalin, followed by embedding in paraffin dispensing module EG 1150H (Leica, Germany). The slices were made utilizing a microtome RM2255 (Leica, Germany), followed by hematoxylin and eosin (H&E) staining.

### 2.12. The Statistical Analysis

Data were statistically analyzed by ANOVA (analysis of variance) using SPSS software (SPSS/24.0; SPSS Inc., Chicago, IL, USA) and the unpaired *t*-test. The value of *p* ≤ 0.05 was significantly considered. The results were presented as the mean ± S.E.M. of the three independent experiments of each test.

## 3. Results and Discussion

### 3.1. Characterization of NPs

**Figure 1:**
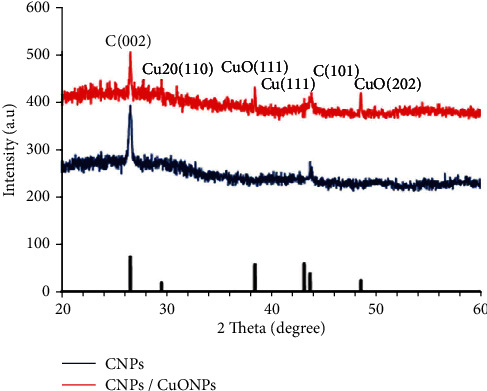
X-ray diffraction (XRD) patterns of the prepared nanoparticles (NPs).

Figure 1 illustrates XRD patterns for CNPs with and without CuO NPs. The diffraction pattern peaks at 26.58° and 43.78° at 2*θ* can be observed belonging to the (002) and (111) plane of the carbon material (JCPDS: 41-1487 & 06-0675) [40]. These data are in agreement with the previously reported results [41]. However, the CNP/CuO NP pattern showed new peaks at 38.5° and 48.64° at 2*θ*, corresponding to the (111) and (202) crystal planes, respectively, of CuO, with a monoclinic phase (JCPDS:05-0661) [42]. This result is consistent with the data previously recorded [43]. Also, additional peaks for Cu2O (110) and Cu (111) were observed for CNP/CuO NP sample at 29.56° (2*θ*) and 43.2° (2*θ*), respectively [44]. The noticeable peaks of the Cu2O (110) plane and Cu (111) may be due to the mechanism of interaction. When the time is shorter, and the temperature is high, it is hard to create CuO completely. So, further oxidation to Cu2O was obtained. It suggests that obtained Cu NPs are less oxidized due to the mechanism of NPs formation. In pulsed nanosecond duration, the ablated material passes through a series of processes: melting, vaporizing, and growing in the plasma due to the interaction with liquid and by coalescence in the surrounding environment [30].

**Figure 2:**
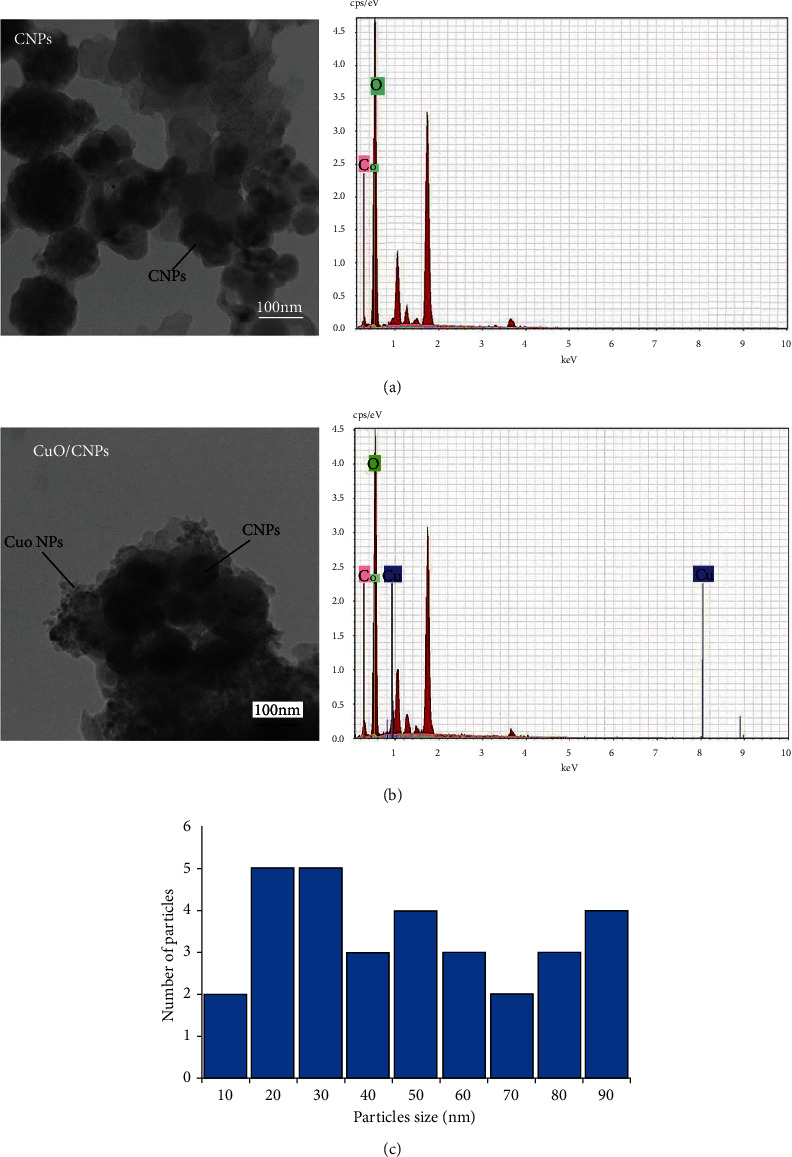
Transmission electron microscope (TEM) images and energy dispersive spectroscopy (EDS) spectra of prepared (a) carbon NPs (CNPs), (b) CNP-copper oxide (CuO) NPs, respectively, and (c) distribution of CNP-CuO NP size regarding particle numbers.

Figure 2 shows TEM images and the size distribution of prepared NPs. From the images, an aggregation was observed, owing to the attractive force among the NPs [45]. It is known from the LAL method that embracing liquid causes a strong impact of confinement, limiting the expansion, thereby causing a high dense species in the plasma, associated with a vast reduction in the temperature. Therefore, these species' diffusion can result in collisions, collections, and producing new molecules [46]. The obtained CNPs have an almost spherical shape with particle sizes ranging between 5 and 40 nm. The chemical composition of the CNPs was characterized using EDS measurements as shown in Figure 2, which specified the existence of C and oxygen (O) in the colloid. It also represents the EDS spectrum of CNP-CuO NPs, which has set the presence of Cu, C, and O in the sample.  Figure 2(c) shows the particle size distribution for CNP-CuO NPs, and the particle size was determined and found from 10 to 90 nm.

### 3.2. CNPs and CNP/CuO NPs as an Antimicrobial Agent

**Figure 3:**
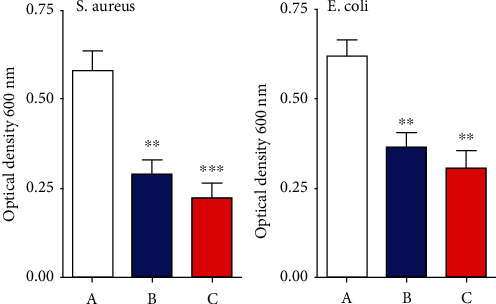
Effect of nanoparticles in bacterial growth. (a) Control-untreated bacterial strain. (b) Treated bacterial strains with CNPs. (c) Treated bacterial strains with CNP/CuO NPs. Values are indicated as mean ± SEM. ^∗∗^*p* < 0.01 and ^∗∗∗^*p* < 0.001.

In this study, we investigated the impact of NPs on the bacterial growth curve. The CNPs and CNP/CuO NPs demonstrated a significant inhibitory effect on *E. coli* and *S. aureus* growth, compared to the untreated strain, especially after 12 h of treatment (Figure 3). The inhibitory effect of CNP/CuO NPs, even though not significant, was observed to be more than that of CNPs.

### 3.3. CNPs and CNP/CuO NPs Increase the Cellular Material Release

**Figure 4:**
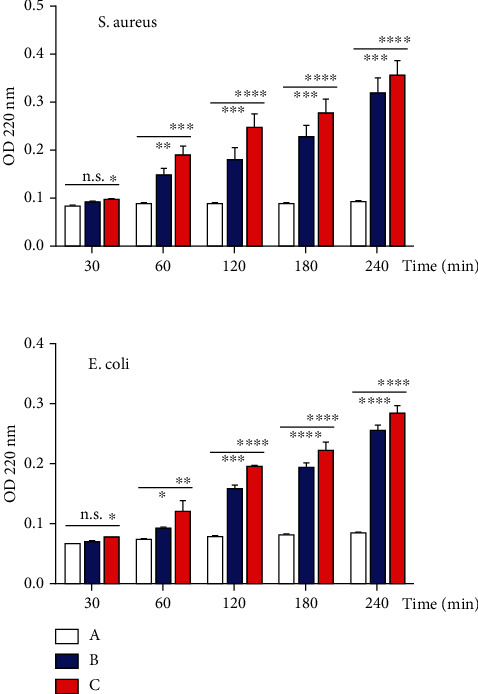
Cellular material release of *E. coli* and *S. aureus* after treatment with prepared NPs. (a) Control-untreated bacterial strain. (b) CNP-treated bacterial strains. (c), CNP-CuO NP-treated bacterial strains.

Absorbing 220 nm of cellular material from *E. coli* and *S. aureus* organism treated with CNPs and CNPs decorated with CuO NPs was analyzed by UV-vis spectroscopy (Figure 4). The optical density at 260 nm of bacterial cells exposed to the prepared NPs displayed a significant increase in the cellular material release in a time-dependent manner. As shown in Figure 4, the effect of CNPs decorated with CuO NPs was higher than that of CNPs for both bacterial strains. Optical density at 260 nm was used to evaluate the leakage of intracellular materials, including nucleic acid, due to the disruption of the cytoplasmic membrane. These results proved that NPs acted on the cytoplasmic membrane, increasing the membrane's permeability and cell death.

### 3.4. Bacteria Strains Shape

**Figure 5:**
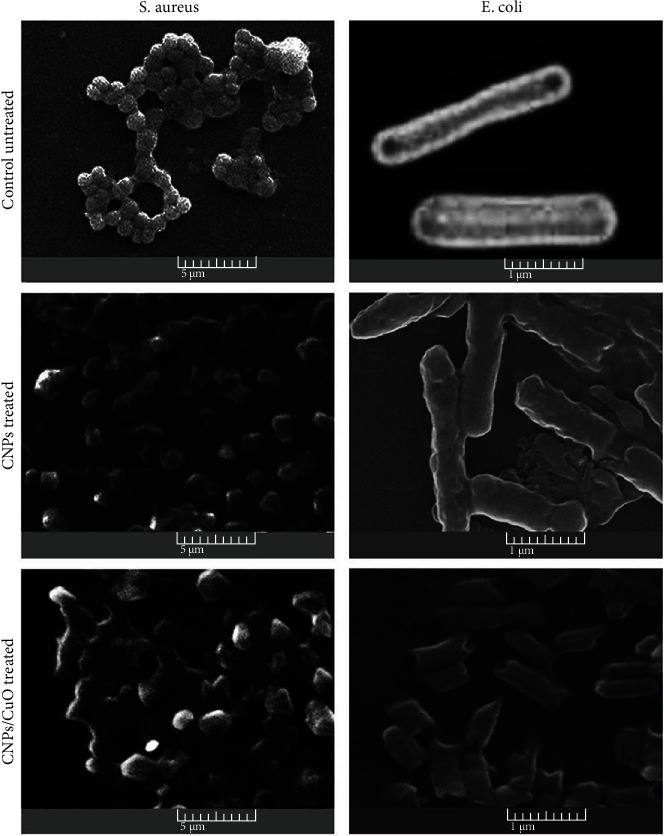
Scanning electron microscope (SEM) images of untreated and treated *E. coli* and *S. aureus* with NPs demonstrate the bacterial cell wall morphology deformation and aggregation of bacteria together.

The shape of *E. coli* and *S. aureus* bacteria was evaluated using the SEM images before and after treating with NPs (Figure 5). The SEM image of control *E. coli* showed uniform bacteria cell surface compared to the treated bacteria cell with typical rod shape, while SEM images of the control *S. aureus* showed a grape-like cluster shape. SEM images of treated bacteria cells with NPs for both bacterial strains indicated little structural changes in cell membranes. These images confirmed the morphological damage in the bacterial cell membranes, making the NPs more penetrable, leading to the release of intracellular materials. The SEM images of treated bacteria cells with carbon NPs decorated with CuO NPs are shown in Figure 5. The effect of decorated NPs on both bacterial strains was clear by deformation the morphology of bacterial cell wall and the aggregation of bacteria together. The NPs affect the outer membrane of the tested microorganisms, as was confirmed by more pores in the cell membrane of the bacterial strains treated NPs. The damage due to osmotic imbalance led to more leakage bacterial cells [47] and the consequential changes in the normal form, osmotic balance, and structural integrity of the cells after exposure to NPs. The SEM images demonstrated more aggregation and membrane rupture of the strains after treating NPs than untreated strains. Furthermore, there was a neutralized bacterial membrane surface potential, increasing the surface tension and the subsequent abnormal structures, rupture, damage, and blebs observed in the bacterial cell membrane [48].

### 3.5. CNPs and CNP-CuO NPs Induce Death of Bacterial Strains

**Figure 6:**
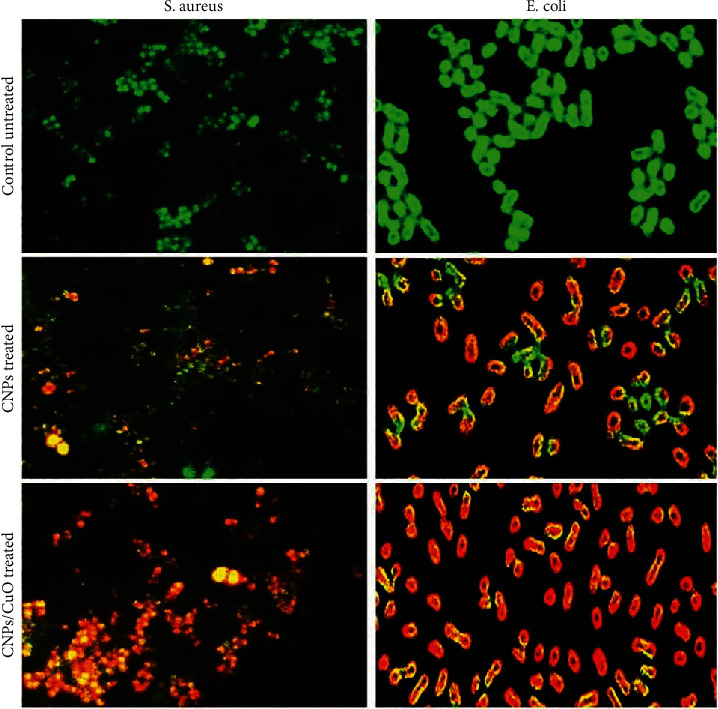
Fluorescence images of *E. coli* and *S. aureus* before and after treatment with CNPs and CNP-CuO NPs. Magnification 100x.

The antibacterial activity of prepared NPs on *E. coli* and *S. aureus* bacterial strains was determined by fluorescence microscopy technique using AO/EtBr dual staining (Figure 6). AO binds to the nucleic acid of living cells and gives a green fluorescence, while EtBr binds only to the dead cell nucleic acid and shows red or orange fluorescence [11]. All untreated bacterial strains showed green fluorescence as an indication of the living state of bacteria for both types of bacteria strains, while most of the bacterial strains treated with carbon and carbon decorated with CuO NPs exhibited red color, indicating the death of the majority of both bacterial strains. Therefore, carbon decorated with CuO NPs has the highest effect on both types of bacterial strains. The impact of NPs on *S. aureus* was higher than on *E. coli* due to the structural difference in the cell membranes. Bacteria DNA damage induced by nanomaterials can be caused by several sources, endogenous or exogenous damages. The endogenous source can occur by the ROS attack. ROS are formed in the cells from the natural cellular metabolism and are very unstable, reacting quickly with other molecules. The chain of reaction between the free radical and DNA generates genotoxic lesions. The external agents may include radiations of ultraviolet, gamma, X-ray, drugs, and nanomaterials, wherein nanomaterials can cause exogenous damage to DNA [49].

### 3.6. Antiproliferative Activity of the CNPs and CNP/CuO NPs

**Figure 7:**
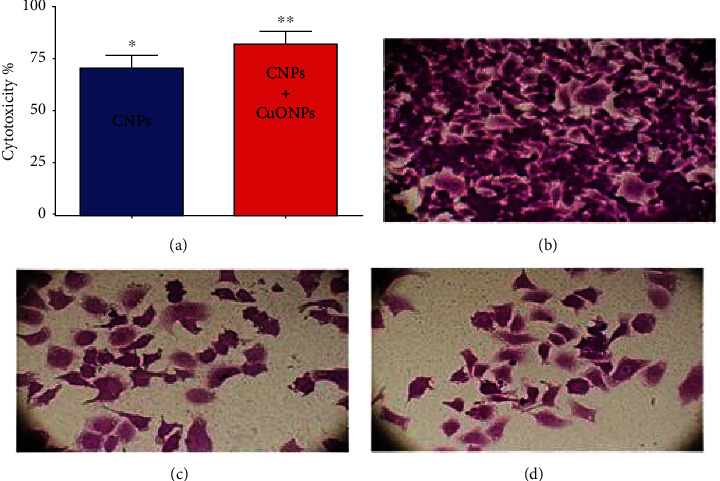
(a) Cytotoxicity activity of CNPs and CNP-CuO NPs against MCF-7 cells. The values represent the mean ± SEM. ^∗^*p* < 0.05 and ^∗∗^*p* < 0.01. The cells were captured using an inverted phase microscope. (b) Control-untreated MCF-7 cells. (c) Cells treated with CNPs. (d) Cells treated with CNP-CuO NPs. Magnification 40x.

The results of the cytotoxic effects and morphological changes in the human cancer cell line (MCF-7) after treatment with CNPs and CNP-CuO NPs for 72 h are displayed in Figure 7; NPs demonstrated significant cytotoxic activity against MCF-7 cell line. CNPs employed cytotoxicity with an inhibition rate almost of 69%, while CNP-CuO NPs showed a higher rate reached 85% (Figure 7(a)). This figure demonstrated changed morphology and the number of MCF-7 cells after treatment with the NPs, confirming that the decorated NPs have the highest impact on the cell due to both NPs' synergistic effects [50, 51]. The untreated cell image showed that the control cell has normal morphology (Figure 7(b)). After adding NPs, MCF-7 cells demonstrated differences in morphology, contact with the surrounding cells, and reduction in their numbers (Figures 7(c) and 7(d)).

### 3.7. NPs Induce Apoptosis in MCF-7 Cells

**Figure 8:**
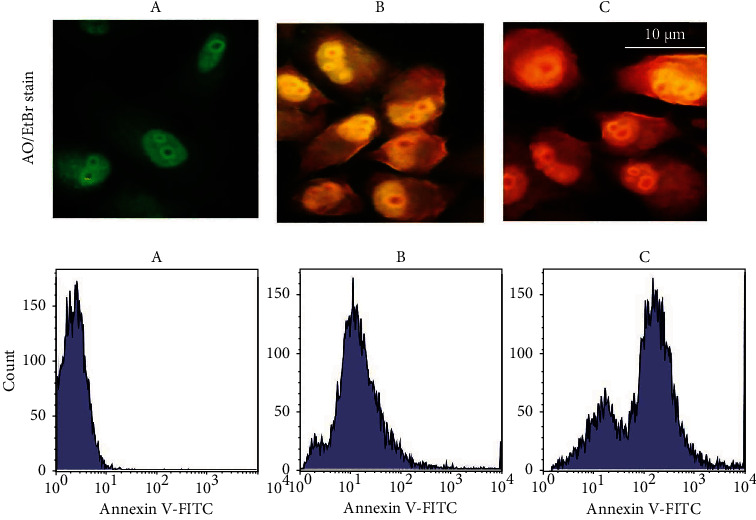
Apoptosis markers in MCF-7 cells following treatment with NPs, upper panel represented orange-ethidium bromide (AO/EtBr) staining, scale bar 10 *μ*m. The lower panel represents the flow cytometry assay. (a) Control-untreated cells, (b) CNP-treated cells, and (c) CNP-CuO NP-treated cells.

Figure 8 exhibits the nucleus morphology of MCF-7 cells treated with NPs. The nonapoptotic cells appeared green by staining with AO, and the apoptotic cells appeared red or orange. Staining was performed with EtBr to recognize the early and late apoptotic cells. The apoptotic cells demonstrated an abnormal nuclear morphology. The NPs might disrupt cell membranes and create vacuoles in the treated cells compared to the control untreated MCF-7 cells, so the copper-based nanostructure could modulate cancer cell metabolism and cause cell death. In addition to the DNA damage due to the ROS formation generated from copper ions produced by dissolution in the medium, these species can form hydroxyl radicals via heterogeneous reactions on the particle. ROS can be the major reason responsible for copper-based nanomaterial toxicity. The percentage of apoptotic cells was determined by staining the MCF-7 cells with the annexin V-FITC using flow cytometry to confirm our results. A sizable increase of apoptosis due to the treatment of the tested cancer cells with NPs is displayed in Figure 8. The present study results showed that the proportion of apoptotic cells was significantly increased compared with the control untreated NPs MCF-7 cells.

### 3.8. Side Effects of NPs In Vivo Study

**Figure 9:**
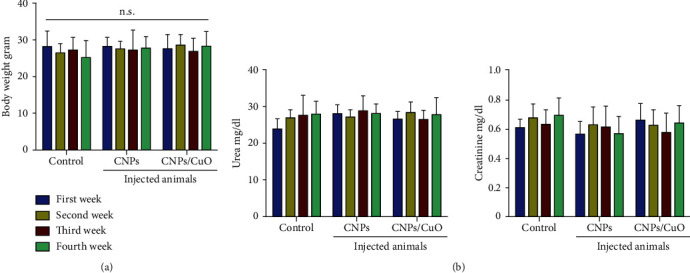
Biocompatibility of CNPs and CNP-CuO NPs; *in vivo* effects of CNPs and CNP-CuO NPs on (a) the body weight, (b) urea, and creatinine.

The animals' body weight was measured in the current study before and after injection with CNPs and CNP/CuO NPs for 4 weeks. The results showed no body weight changes among the injected sets, as shown in Figure 9(a). In addition, Figure 9(b) shows kidney functional parameters examined using biochemical tests after treatment with NPs for four weeks. No significant differences in urea and creatinine levels were observed, indicating no toxic impacts of NPs on renal activity.

**Figure 10:**
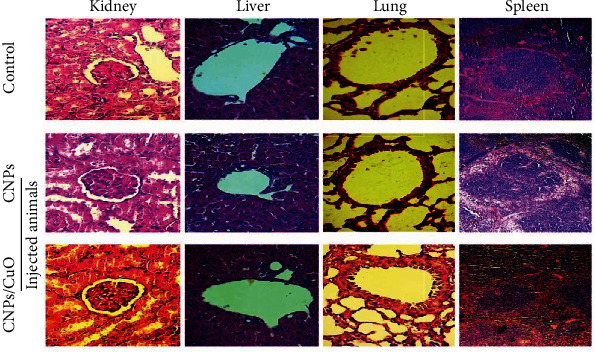
Histological sections of the kidney, liver, lung, and spleen in mice injected with CNPs and CNP-CuO NPs. Magnification power 40x.

Histopathological inspections evaluated the toxic effects of CNPs and CNP-CuO NPs; Figure 10 indicates normal kidney structure without any morphological changes. The liver tissue is also illustrated in Figure 10, showing a normal structure after treatment with NPs. Furthermore, the spleen's lymphoid nodules showed no decrease in the number of lymphocytes. The lung tissues as well showed similar features in both sets Figure 10. The outcomes showed the lung tissue with the ordinary structure without any distinguishable changes between the control and NPs treated sets. As a result, CNPs and CNP-CuO NPs did not display any effect of the histopathological alterations in the treated animals' kidneys, liver, lungs, and spleen. The above results were compared with the previously reported observations in Table 1, which unequivocally showed the effects of the current preparations of CuO NPs and CuO NPs-CNPs. Table 1 provides a listing of the NPs, their characteristics, and their activity.

## 4. Conclusions

A feasible, straightforward, robust, and eco-friendly PLAL technique was successfully utilized to produce CNPs and CuO NPs-CNPs. The decorated structures were characterized by XRD analysis, while the TEM images confirmed the CNP decoration in the colloidal medium with the CuO NPs. The study examined the antibacterial activity of the prepared NPs. The CNP-CuO NPs showed antimicrobial inhibitory activity against both the pathogenic bacterial strains, as well as their high potential as an anticancer agent with higher cytotoxicity against breast cell lines than the CNPs alone. The cytotoxic effect of the CNP-CuO NPs against the MCF-7 cell lines was evident in the MTT assay and AO/EtBr dual staining. The highest anticancer effects reached almost 85% for CNP-CuO NPs. Eventually, the CNPs and CNP-CuO NPs did not display any significant *in vivo* side effects, either indicated by kidney or liver tests. The nanomaterial properties and applications can further be extended to other materials transformations to nanoscale modules of several different metallic and nonmetallic targets and investigate different bioactivities in *in vivo* conditions.

## Figures and Tables

**Table 1:** CuO NPs and CuO/CNPs antimicrobial and anticancer activities.

CuO NPs or modified CuO NPs	Size	Concentrations	Methods	Purpose	Conclusions	References
CuO NPs	577 nm	50-500 *μ*g/ml	Green synthesis from *F. religiosa* leaves	Anticancer activity of human lungs cancer	Cytotoxicity of CuO nanoparticles against human lung cancer (A59) cells augmented with increased dose concentration, whereby it showed 6% viability at higher concentration (500 *μ*g/ml) rather than the 70% viability at the lower concentration of 50 *μ*g/ml.	[52]
CuO NPs	5-15 nm	—	Picosecond laser ablation in air and argon gas	Antimicrobial activity against *S. aureus*	Copper nanoparticles prepared 1064 nm—picosecond laser in argon showed strong antibacterial activity against gram-positive bacteria, S. aureus.	[30]
CuO NPs	30 and 60 nm	0.1 mg/ml	Chemical reduction method to prepare aqueous Cu colloid	Antibacterial and antitumor effects	CuO NPs of both 30 nm and 60 nm sizes demonstrated increased NP concentration causing decreased survival and expression of MMP-2 and VEGF genes and increased apoptosis in 4T1 cancer cells. Furthermore, at all concentrations, CuO NPs of 30 nm equated to 60 nm NPs demonstrated dual impact on anti-cancer and antibacterial activities.	[53]
CuO NPs	20 nm	250, 500, and 1000 *μ*g/ml	Biosynthesized CuO nanoparticles from *Nilgirianthus ciliatus* plant extract	Antibacterial and anticancer activity	CuO NPs exhibited noticeable activity on both the MCF-7 and A549 cancer cell lines and showed minimum cytotoxicity on normal cells, fibroblast (L929).	[54]
CuO NPs/carbon nanocomposites	11-22 nm	0.250 mg/ml, 0.5 mg/ml, and 1.0 mg/ml	Green synthesis method using leaf extracts of *Adhatoda vasica*	Antimicrobial activity (antifungal activity and antibacterial)	CuO/C nanocomposites exhibited remarkable antimicrobial activities against gram-negative bacteria, *P. aeruginosa*, *E. coli*, and *K. pneumoniae*, and the gram-positive bacteria, *S. aureus*, as well as against the fungi, *A. niger* and *C. albicans*.	[55]
CuO NPs decorated CNPs	5-40 nm diameter range	50 *μ*g/ml	Pulsed laser ablation in liquid	Antimicrobial activity against *S. aureus* and *E. coli* and anticancer potential on MCF-7	CNP-CuO NPs showed inhibitory activity against both pathogenic bacterial strains and high potential as an anticancer agent, with higher cytotoxicity against breast cancer cells than the CNPs alone. The highest anticancer activity impact for CNP-CuO NPs reached ~85%. In addition, CNPs and CNP-CuO NPs did not display any significant *in vivo* side effects as indicated by kidneys and liver functions tests.	Current study

CuO: copper oxide; NPs: nanoparticles; CNPs: carbon NPs.

## Data Availability

The data used to support the findings of this study are included within the article.
